# Ca^2+^-Sensitive Potassium Channels

**DOI:** 10.3390/molecules28020885

**Published:** 2023-01-16

**Authors:** Razan Orfali, Nora Albanyan

**Affiliations:** 1 Biomedical Research Administration, Research Centre, King Fahad Medical City, Riyadh 12231, Saudi Arabia; 2 Clinical Pharmacy Administration, King Fahad Medical City, Riyadh 12231, Saudi Arabia

**Keywords:** Ca^2+^-activated potassium channels, K_Ca_ 1.1, K_Ca_2.x and K_Ca_3.1 channels, Ca^2+^ sensitivity, channelopathies

## Abstract

The Ca^2+^ ion is used ubiquitously as an intracellular signaling molecule due to its high external and low internal concentration. Many Ca^2+^-sensing ion channel proteins have evolved to receive and propagate Ca^2+^ signals. Among them are the Ca^2+^-activated potassium channels, a large family of potassium channels activated by rises in cytosolic calcium in response to Ca^2+^ influx via Ca^2+^-permeable channels that open during the action potential or Ca^2+^ release from the endoplasmic reticulum. The Ca^2+^ sensitivity of these channels allows internal Ca^2+^ to regulate the electrical activity of the cell membrane. Activating these potassium channels controls many physiological processes, from the firing properties of neurons to the control of transmitter release. This review will discuss what is understood about the Ca^2+^ sensitivity of the two best-studied groups of Ca^2+^-sensitive potassium channels: large-conductance Ca^2+^-activated K^+^ channels, K_Ca_1.1, and small/intermediate-conductance Ca^2+^-activated K^+^ channels, K_Ca_2.x/K_Ca_3.1.

## 1. Introduction

Ca^2+^ is essential for the survival and functioning of all living cells [1]. It is an important signaling molecule that crosses membranes, acting as a homeostatic regulator for both intracellular and extracellular fluid [2]. Ca^2+^ regulates a wide range of functions in all types of cells, involving several ion channels [1]. Ca^2+^ ions offer ultimate properties in size, charge, availability, and ionization potential, allowing them to flow rapidly, yet simultaneously, bind deeply. Thus calcium might be considered the prominent intracellular signaling molecule, as it is involved in numerous physiological processes such as neurotransmission, muscle contraction, the regulation of gene expression, fertilization, and mitosis [3,4,5]. Unique among these Ca^2+^ regulatory processes are the Ca^2+^ sensitive ion channels or Ca^2+^-activated ion channels. The gating of these channels is modulated by Ca^2+^-sensing proteins such as calmodulin CaM [3,6]. CaM is characterized by its ability to bind and release Ca^2+^ over the physiological Ca^2+^ concentrations. It undergoes a significant conformational change in Ca^2+^ binding and consequently regulates these specific ion channels to modify their function. Therefore, the physiological roles and functions of these channels can be affected by changes in intracellular Ca^2+^ concentration. Some Ca^2+^-sensitive ion channels include Ca^2+^-activated anion (Cl^−^) channels, Ca^2+^-regulated nonselective cation channels, and Ca^2+^-activated K^+^ channels. Here we will elaborate on the Ca^2+^-sensing mechanisms of the Ca^2+^-activated K^+^ channels.

Potassium channels are the most diverse and abundantly expressed ion channels in living organisms. These channels are expressed in the most excitable and non-excitable cells. They perform various vital functions and can be classified into several different groups. Among these groups are the Ca^2+^-activated potassium channels (K_Ca_ channels), a large family of potassium channels activated by rises in cytosolic Ca^2+^ [7,8]. Over the last two decades, advances have been made in K_Ca_ channel research, including the channels’ functions, expression, pharmacology, and genetic mutations associated with channelopathies. The family of K_Ca_ channels shares a typical functional role by coupling the increase in intracellular Ca^2+^ concentration to the hyperpolarization of the membrane potential. Thus, K_Ca_ channels are critical for maintaining K^+^ homeostasis and cell volume. Additionally, K_Ca_ channels modulate several physiological processes, from neuron firing properties to transmitter release control [8].

K_Ca_ channels can be divided into three main subfamilies based on their single-channel conductance, SK or K_Ca_2 (small conductance; ∼4–14 pS), IK or K_Ca_3.1 (intermediate conductance; ∼32–39 pS), and BK or K_Ca_1.1 (large conductance; ∼200–300 pS) channels [8,9,10]. K _Ca_1.1 channels are activated by intracellular Ca^2+^ and membrane voltage synergistically. However, K _Ca_2x and K _Ca_3.1 are gated solely by internal Ca^2+^ ions and are more sensitive to Ca^2+^ than K _Ca_1.1 channels. K_Ca_2 includes three small-conductance K_Ca_2-channel subtypes, K_Ca_2.1 (SK1), K_Ca_2.2 (SK2), and K_Ca_2.3 (SK3) [8,11,12]. The Ca^2+^-binding sites and Ca^2+^-binding affinities are significantly different among these channel groups. K_Ca_1.1 directly binds Ca^2+^ and has a very low Ca^2+^-binding affinity associated with the regulator of conductance of K+ (RCK) Ca^2+^-binding domains (1–11 μM) [7,8]. In contrast, K_Ca_2.x and KCa3.1 channels share the calmodulin-mediated gating mechanism with a high Ca^2+^-binding affinity due to the constitutive binding of calmodulin (300–600 nM) [13,14,15]. K_Ca_1.1 and K_Ca_2/3 channels show minimal sequence homology. The latter’s S4 segment comprises fewer charged residues than the S4 segment of K_Ca_1.1, which results in a lack of voltage dependence in K_Ca_2/3 channels, enabling them to remain open at negative membrane potentials and thus hyperpolarize the membrane toward values near the K^+^ equilibrium potential [11,14]. Each type of K_Ca_ channel shows distinct pharmacology, and the activity of each hyperpolarizes the membrane potential. Here we will discuss these unique potassium ion channels and their sensitivity to Ca^2+^, aiming to understand the mechanisms behind Ca^2+^-dependent regulation.

## 2. Ca^2+^-Activated K_Ca_1.1 Channels

Large-conductance Ca^2+^-activated K^+^ (BK_Ca_) channels are activated under dual control by both calcium and the membrane voltage (membrane depolarization). The calcium dependence of these channels changes steeply according to membrane potentials, with the calcium Kd in the micromolar range at resting membrane potentials (~–60 mV) but in the nanomolar range at depolarized potentials (+20 to +40 mV). Remarkably, these channels can open in the absence of calcium, and it seems that the effects of calcium and membrane potential are independent processes that both increase the open probability. While the voltage dependence of these channels is quite weak compared to the other purely voltage-gated potassium channels, the voltage dependence of K_Ca_1.1 enables them to act as coincidence detectors, which is central to their physiological function [7]. These channels are expressed on the plasma membrane as tetramers of α subunits. The gene encoding the α subunit (slo; *KCNMA1*) of K_Ca_1.1 channels (K_Ca_1.1; slo1) was cloned in the early 1990s from *Drosophila*. K_Ca_1.1 channels are widely distributed in various cells and regulate Ca^2+^ influx and many Ca^2+^-dependent physiological processes [8,16,17].

### 2.1. Expression and Physiology of K_Ca_1.1 Channels

In neurons, the activation of K_Ca_1.1 channels repolarizes the membrane and reduces Ca^2+^ influx into the cells [7,18,19]. K_Ca_1.1 channels participate in numerous physiological processes via the modulation of membrane excitability and Ca^2+^ homeostasis [18]. Examples include modulating neurotransmitter release, regulating vascular and respiratory tone, neurovascular coupling [7], endocrine secretion, and urinary bladder tone.

K_Ca_1.1 channels control hormonal secretion by altering the duration and frequency of action potentials [20,21]. The main functions of K_Ca_1.1 channels in neurons are to generate fast afterhyperpolarization after an action potential [22]. In neurovascular coupling, K_Ca_1.1 channels umpire most of the dilation and the entire vasoconstriction in astrocytic end feet [18,23]. In retinal circulation, K_Ca_1.1channels are contributed to the regulation of retinal blood flow via the action of several vasodilators in the endothelium and vascular smooth muscle. A recent study has shown that administering a K_Ca_1.1 channel activator (BMS-191011) to male Wistar rats improved retinal circulation [18,24,25]. In the urinary system, K_Ca_1.1 channels have been reported in various renal cell types, such as urinary bladder smooth muscle cells [26], glomerular mesangial cells [27], afferent arterioles [28], and podocytes in the Bowman’s capsule [29].

It has been demonstrated that K_Ca_1.1 channels provide negative feedback, preventing contractions induced by agonists. Moreover, several segments of the distal convoluted tubules of the nephron have been shown to express K_Ca_1.1 channels that are suggested to contribute to the volume regulation in the distal convoluted tubules of the nephron [26,28].

**Figure 1:**
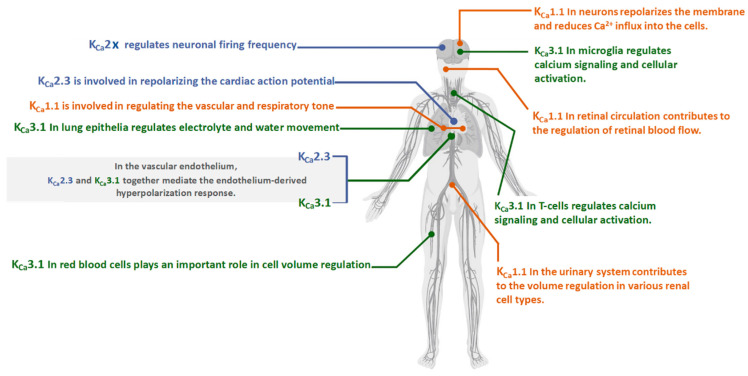
Expression and Physiology of the K_Ca_2x, K_Ca_3.1, and K_Ca_1.1 channels [10].

The list continues with numerous studies identifying enormous regulatory physiological mechanisms. K_Ca_1.1 channels may serve as a potential target (Figure 1).

### 2.2. K_Ca_1.1 Channels Structure

K_Ca_1.1 channels are composed of four pore-forming subunits (α) encoded by the *Slo1* gene or *KCNMA1* in mammals. The *Slo1* gene undergoes alternative splicing leading to a high degree of functional diversity in the K_Ca_1.1 channels [16,30]. Each α (*Slo1*) subunit contains three main domains: a voltage sensor domain (S0–S4), a pore-gate domain (S5–S6), and a long intracellular C-terminal cytosolic region, which functions as a Ca^2+^ sensor domain. K_Ca_1.1 channels are also co-assembling and prominently impacted by diverse auxiliary subunits, including β (β1-4), γ (γ1-4), and LINGO1 subunits that give rise to the existence of distinct K_Ca_1.1 channel phenotypes with diverse functionality [16,29,31,32].

The Ca^2+^ sensor domain comprises two non-identical domains (i.e., RCK1 and RCK2), which contain high-affinity binding Ca^2+^ sites (Ca^2+^ bowl) that have been implicated in the direct gating of the channel [16,33,34]. The K_Ca_1.1-β-subunits have been shown to affect the Ca^2+^ sensitivity of the K_Ca_1.1 channel gating [35,36]. Thus, due to these structural features, the K_Ca_1.1 channel is characterized by unique biophysical properties, including ion permeation, gating, and modulation by diverse ligands and intracellular molecules.

**Figure 2:**
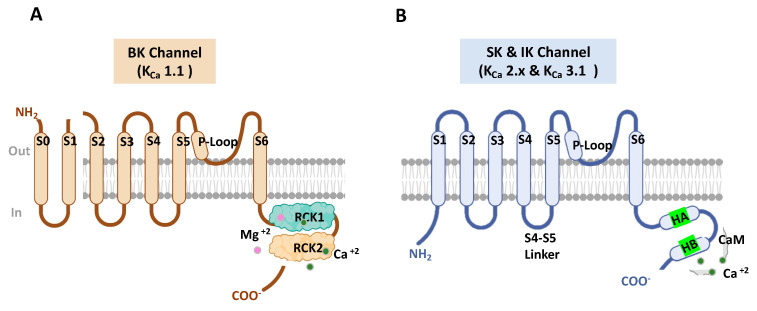
Diagram of the general K_Ca_1.1 and K_Ca_2.x channel structure. (**A**) Schematic channel topology of one K_Ca_ 1.1 α-subunit, including the pore-gate domain between (S5–S6) and a C-terminal cytosolic region that functions as a Ca^2+^ sensor constituted by two non-identical domains (RCK1 and RCK2), which contain high-affinity binding Ca^2+^ sites (Ca^2+^ bowl) and several domains for multiple ligands or cations such as Mg^2+^. (**B**) Schematic channel topology of one K_Ca_2.x α-subunit, including the CaM that serves as the K_Ca_2.x and K_Ca_3.1 channel’s Ca^2+^ sensor. A and B were generated using Biorender.com.

Cryogenic electron microscopy (cryo-EM) [37] has begun to provide crucial structural and biophysical insights into the K_Ca_1.1 channel gating. Structure–function studies of K_Ca_1.1 channels are needed to provide a better understanding of these channels in search of novel compounds to treat diverse K_Ca_1.1-associated pathologies [17,38] (Figure 2A).

### 2.3. Channelopathies of K_Ca_1.1 Channels

Channelopathies are conditions caused by pathogenic alterations in ion channel activity that disrupt homeostasis and physiological functions [39,40]. With advances in whole-exome sequencing (WES), various monogenetic channelopathies are now diagnosable. However, the molecular basis of these mutations and how they produce clinical phenotypes remains unclear. Because of the small number of patients and the lack of genetic pedigree analysis, we have a limited understanding of channelopathies [41]. Alterations in the activity of K_Ca_1.1 channels were demonstrated in several channelopathies. Human *KCNMA1* mutations are primarily linked with neurological conditions, such as seizures, developmental delay, movement disorders, and intellectual disability. *KCNMA1* mutations are also associated with several other pathologies, including diabetes [42], atherosclerosis [43], hypertension [44], and cardiac hypertrophy [45]. These mutations involve gain-of-function (GOF) and loss-of-function (LOF) alterations in K_Ca_1.1 channel activity, together with several variants of unknown significance (VUS). Evidence suggests that the mutation alterations of the K_Ca_1.1 channel may associate with semi-distinct patient symptoms, such as paroxysmal nonkinesigenic dyskinesia (PNKD) with GOF and ataxia with LOF. K_Ca_1.1 channel dysfunction can also lead to urinary incontinence and an overactive bladder [46]. Data has already suggested that the deletion of the K_Ca_1.1 gene has been associated with progressive hearing loss [47]. Other results [48] indicated that losing the K_Ca_1.1 channel leads to erectile dysfunction. Though most *KCNMA1* mutations are de novo in origins, additional evidence is needed to establish causality in most cases. Additionally, GOF and LOF in K_Ca_1.1 channels are linked with overlapping symptoms. However, it is unclear whether selective agonists or antagonists that correct the level of K_Ca_1.1 channel activity will produce the desired outcome on neuronal activity [39,40].

## 3. Ca^2+^-Activated K_Ca_ 2.x Channels

K_Ca_2.x channels are members of the voltage-insensitive calcium-activated potassium channel family that are stimulated by the elevation of the cytosolic calcium concentration. Upon activation, K_Ca_2.x channels allow K^+^ ions to leave the cell as a function of the difference between the depolarized cell and the K^+^ equilibrium potentials [49]. K_Ca_2.x subunits are encoded by the *KCNN1* (K_Ca_2.1; SK1), *KCNN2* (K_Ca_2.2; SK2), and *KCNN3* (K_Ca_2.3; SK3) genes [50], while IKα (K_Ca_3.1; SK4) are encoded by the *KCNN4* gene [51].

### 3.1. Expression and Physiology of K_Ca_ 2.x Channels

The three subtypes of K_Ca_2.x channels (K_Ca_2.1–3) are expressed in many areas of the central nervous system and are involved in afterhyperpolarization upon activation. In the cerebellum, K_Ca_2.2 is the primary channel subtype expressed in Purkinje cells and has a crucial role in the Purkinje cell peacemaking [52,53]. Furthermore, K_Ca_2.x channels are expressed on the neuronal plasma membrane and are suggested to exert neuroprotective effects by modulating the firing pattern of dopaminergic neurons. In dopaminergic neurons, K_Ca_2.x channels were determined on the membrane of mitochondria and, upon activation, were suggested to prevent mitochondrial dysfunction [54]. In the cardiovascular system, K_Ca_2.x subtypes are expressed in the atrial and ventricular walls and play essential roles in regulating the activity of atrial myocytes. K_Ca_2.3 and K_Ca_3.1 are predominant subtypes expressed in endothelial cells and play a crucial role in vasodilation, which is mediated by an endothelium-derived hyperpolarizing factor (EDHF) [55,56]. The diversity of K_Ca_2.x physiological roles are widened due to numerous splicing variants in different tissues (Figure 1) [57].

### 3.2. K_Ca_ 2.x Channels Structure

All three K_Ca_2.x subtypes and K_Ca_3.1 form tetrameric channels and are between 553 and 580 amino acids long. The α subunits of these channels comprise six transmembrane helices (S1–S6), the pore region between helices S5 and S6), and cytosolic N- and C-terminal domains. Their biophysical characteristics are their independence from membrane voltage because they have lost most of the positively charged residues commonly associated with voltage-dependent gating [58]. Low intracellular Ca^2+^ concentrations activate K_Ca_2.x channels through a unique calmodulin (CaM) gating mechanism. (Figure 2B).

### 3.3. Channelopathies of K_Ca_ 2.x Channels

The discovery of mutations in genes encoding K^+^ channel subunits has provided unique insights into the pathophysiology of human disorders affecting the central nervous system, heart, kidney, and other organs [59]. Decreased or increased activity of K_Ca_ channels caused by loss-of-function (LOF) and gain-of-function (GOF) variants in the corresponding genes, respectively, underlies a broad spectrum of human channelopathies [60,61]. There are no known genetic disorders associated with K_Ca_2.1 channels in humans. While neurodevelopmental disorders such as cerebellar ataxia and tremor are associated with loss-of-function K_Ca_2.2 mutations [59]. The GOF mutations of K_Ca_2.3 are linked with the Zimmermann–Laband syndrome (ZLS) [60,62,63,64] and have also been associated with an idiopathic non-cirrhotic portal hypertension (INCPH) [60,62,65], In one study [66], CAG repeat polymorphism in *KCNN3* was linked to schizophrenia. On the other hand, another study [67] found that a mutation of the K_Ca_2.3 channel gene (*L283fs287X*) results in the deletion of the protein N-terminal region identified in schizophrenic patients. These resulting mutant K_Ca_2.3 channels were found to dominantly suppress K_Ca_2.3 channel currents [67] (LOF mutation). It has been reported that GOF mutations of K_Ca_3.1 are linked with a subset of hereditary xerocytosis (HX) (OMIM 194380), which is an autosomal dominant congenital hemolytic anemia characterized by erythrocyte dehydration [60,68,69].

## 4. Ca^2+^- Sensitivity of K_Ca_ Channels

The intracellular Ca^2+^ concentration and membrane potential are important metabolic parameters for all organisms. Ca^2+^ is involved in numerous physiological processes, including muscle contraction, the regulation of gene expression, neurotransmission, mitosis, and fertilization.

Ca^2+^-sensitive ion channels allow for crosstalk between chemical and electrical systems and the feedback control of Ca^2+^ entry into the cells [5,10]. The unique Ca^2+^-activated potassium channels are best known for enabling changes in intracellular Ca^2+^ concentration to influence neuronal firing patterns and the strength of muscle contraction. K_Ca_2.x/K_Ca_3.1 are voltage-independent and are activated by low intracellular Ca^2+^ concentrations. Remarkably, K_Ca_ 2.x and K_Ca_3.1 display similar Ca^2+^ sensing properties (Ca^2+^ dose-response relationships) and respond rapidly to changes in Ca^2+^ with time constants of 5–15 ms [13].

Ca^2+^-binding protein calmodulin (CaM) serves as the K_Ca_2.x and K_Ca_3.1 channel’s Ca^2+^ sensor [10,70] (Figure 2B). K_Ca_1.1 channels are both Ca^2+^ and voltage-activated, and their sensitivity to voltage and intracellular Ca^2+^ are prominently influenced by their association with auxiliary and non-pore-forming modulatory β (β1-4), γ (γ1-4), and LINGO1 subunits [32,71,72]. Site-directed-mutagenesis experiments have led to understanding the K_Ca_1.1 channel’s Ca^2+^ binding sites. The K_Ca_1.1 structure (Figure 2A) displayed shows that each α subunit contains a pair of RCK domains in the C-terminus portion where the Ca^2+^ bowl resides. The K_Ca_1.1 Ca^2+^ gating interface is made by the intersubunit assembly interface, and mutational analyses studies of the putative interacting residues in human BK channels discovered that E955 is a determinant of Ca^2+^ sensitivity through intersubunit electrostatic interactions. These findings proved that the intersubunit assembly interface contains molecular determinants of Ca^2+^ sensitivity in K_Ca_1.1 channels [73].

**Figure 3:**
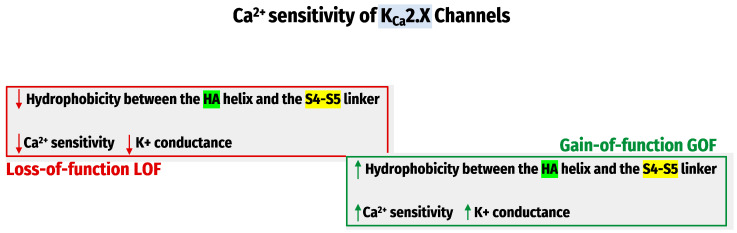
The regulation of K_Ca_2.X Channels’ Ca^2+^ sensitivity.

Recently, the Cryo-electron-microscopy structures of the entire K_Ca_1.1 channel [74] have significantly advanced our understanding of Ca^2+^ sensing mechanisms. Therefore, a tremendous effort has been devoted to developing small molecules targeting K_Ca_ channels. These small molecules could modulate K_Ca_ channels positively or negatively by influencing the apparent Ca^2+^ sensitivity of these channels. We have recently investigated how the K_Ca_2.x channel’s apparent Ca^2+^ sensitivity is regulated and found that the hydrophobic interactions between the HA helix and S4-S5 linker regulate the K_Ca_2.x channel’s apparent Ca^2+^ sensitivity. The methods utilized were site-directed mutagenesis, patch-clamp recordings, and molecular dynamic (MD) simulations. The observations determined that mutations that decrease hydrophobicity at the HA-S4–S5 interface led to Ca^2+^ hyposensitivity of K_Ca_2.x channels. The mutation that increases hydrophobicity results in hypersensitivity to Ca^2+^ [75] (Figure 3). These studies are based on the apparent Ca^2+^ sensitivity of the amino acid sequences of K_Ca_2.x, which disclosed several differences between their channel subtypes. More studies are needed for the potential drug development for channelopathy disorders.

Genetic mutations encoding K_Ca_ channels change their apparent Ca^2+^ sensitivity and lead to subsequent LOF, a decrease in Ca^2+^ sensitivity, or GOF, and Ca^2+^ hypersensitivity of these channels have been directly linked to many human diseases. Loss-of-function K_Ca_2.2 mutations are associated with neurodevelopmental disorders, such as ataxias and tremors [76,77]. Gain-of-function of K_Ca_2.3 mutations is associated with the Zimmermann–Laband syndrome (ZLS) [78,79] and idiopathic non-cirrhotic portal hypertension (INCPH) [80]. In contrast, GOF K_Ca_3.1 mutations are linked with hereditary xerocytosis (HX) [81].

Human *KCNMA1*-linked channelopathy mutations are primarily associated with neurological conditions, including movement disorders, seizures, and intellectual disability. These mutations comprise GOF and LOF alterations in K_Ca_1.1 channel activity and some variants of unknown significance (VUS). Over the past decade, a great effort has been devoted to the Ca^2+^ sensing mechanisms of Ca^2+^-activated K^+^ channels. Each channel type uses a unique molecular mechanism to regulate its Ca^2+^ gating. Site-directed mutagenesis, in silico modeling, and electrophysiology have proven to make powerful tools in studying Ca^2+^-dependent gating that leads to the development of more selective biophysical and pharmacological approaches for the K_Ca_ channelopathy.

The hydrophobic interactions between the HA helix and S4–S5 linker regulate the K_Ca_2.x channel’s apparent Ca^2+^ sensitivity.

## 5. Summary

Sensitive K_Ca_ channels reviewed as Table 1.

## Figures and Tables

**Table 1:** Sensitive K_Ca_ channels reviewed.

	K_Ca_1.1	K_Ca_2.x	K_Ca_3.1
Expression	Neurons [7,18,19] vascular, respiratory, endocrine, retinal circulation [26], glomerular mesangial cells [27], and podocytes [29].	Neurons, heart, and vascular endothelium [57].	Microglia, lung epithelia, GI epithelia, T cells, and red blood cells [57].
Mechanism	Activated by both Ca^2+^ and Voltage [18].	Activated by low intracellular Ca^2+^ concentrations (Voltage-independent).	
Ca^2+^ binding sites	Two intracellular Ca^2+^-sensing RCK domains (RCK1 and RCK2) [16].	Ca^2+^- binding (CaM) [10,70].	
Ca^2+^ sensitivity Regulation	The inter-subunit assembly interface contains molecular determinants of Ca^2+^- sensitivity in K_Ca_1.1 channels [73].	Hydrophobic interactions between the HA helix and S4-S5 linker regulate the K_Ca_2.x/K_Ca_3.1 channel’s apparent Ca^2+^ sensitivity [75].	
Channelopathies	Neurological disorders, diabetes [42], atherosclerosis [43], hypertension [44], cardiac hypertrophy [45], paroxysmal nonkinesigenic dyskinesia (PNKD), ataxia, hearing loss [47], urinary incontinence and overactive bladder [46].	Cerebellar ataxia and tremor are associated with LOF K_Ca_2.2 mutations [59]. The GOF mutations of K_Ca_2.3 are linked with (ZLS) [60,62,63,64] and (INCPH) [60,62,65], and schizophrenia [66].	The GOF mutations of K_Ca_3.1 are linked with a subset of (HX) [68].

## Data Availability

Not applicable.
